# Novel natural killer cell-mediated cancer immunotherapeutic activity of anisomycin against hepatocellular carcinoma cells

**DOI:** 10.1038/s41598-018-29048-8

**Published:** 2018-07-13

**Authors:** Miok Kim, Seon-Jin Lee, Sangsu Shin, Kang-Seo Park, Sang Yoon Park, Chang Hoon Lee

**Affiliations:** 1 Bio & Drug Discovery Division, Center for Drug Discovery Technology, Korea Research Institute of Chemical Technology (KRICT), Daejeon, 305-600 Republic of Korea; 2 Immunotherapy Convergence Research Center, Korea Research Institute of Bioscience & Biotechnology (KRIBB), Daejeon, 34141 Republic of Korea; 3 Department of Animal Biotechnology, Kyungpook National University, Sangju, Gyeongbuk 37224 Republic of Korea; 4 Department of Oncology, Asan Medical Center, University of Ulsan College of Medicine, Seoul, 05505 Republic of Korea

## Abstract

Despite advances in the clinical management of hepatocellular carcinoma (HCC), this form of cancer remains the second leading cause of cancer-related death worldwide. Currently, there are few treatment options for advanced HCC. Therefore, novel treatment strategies for HCC are required. Here, we described the promising antitumour effects of anisomycin, which exerts both direct killing effects and natural killer cell (NK)-mediated immunotherapeutic effects in HCC. To better elucidate the mechanisms through which anisomycin mediates its antitumour effects, we performed a genome-scale transcriptional analysis. We found that anisomycin treatment of HCC differentially modulated a broad range of immune regulation-associated genes. Among these immune regulation-associated genes, we found that lymphocyte function-associated antigen-3 (LFA-3, also called *CD58*), whose expression was significantly increased in anisomycin-treated HCC cells, was a critical player in NK-mediated immunotherapeutic effects. Furthermore major histocompatibility complex molecules class I (MHC-I) on HCC cells were also significantly regulated by treatment of anisomycin. Those adhesion molecules like CD58, MHC-I, and ICAM4 should be important for immune synapse formation between NK cells and HCC cells to boost NK-mediated immunotherapeutic effects. Notably, this is the first report of NK-dependent immunomodulatory effects of anisomycin suggesting anisomycin as a novel therapeutic drug for treatment of HCC.

## Introduction

Hepatocellular carcinoma (HCC) is the fifth most common malignancy and the second leading cause of cancer-related death worldwide^1^. Many efforts have been made to improve the clinical management of patients with HCC; however, the current prognosis of HCC is poor, primarily due to high metastasis and recurrence rates, leading to low overall survival^2^. The lack of effective therapeutic agents necessitates the urgent development of novel drugs for the treatment of advanced HCC.

Recent advances in the tumour immunology field have suggested that immune cells are critical players in the development, metastasis, and recurrence of multiple types of tumours^3–6^. Based on several recent reports^7–9^, various immune cells, such as T cells, natural killer (NK) cells, macrophages, dendritic cells (DC), and myeloid-derived suppressor cells (MDSC), are present in tumour tissues of patients with HCC and interact with tumour cells. Notably, the antitumoral capacity of tumour-associated immune cells is functionally suppressed by multiple regulatory molecules in the tumour microenvironment, and tumour cells are equipped with immune escape mechanisms^10^. Thus, the development of therapeutic strategies requires the restoration of the antitumoral effects of tumour-associated immune cells and inhibition of the immune escape mechanisms of tumour cells. Specifically, the antitumour killing capacity of NK cells, which are known for their potent tumour cell-killing effects, is reported to be suppressed in multiple tumours, including HCCs^11^. Tumour cells use various strategies for this immune suppression or escape: for example, multiple types of tumour cells suppress the expression of NK group D2 (NKG2D) ligands, such as UL16 binding protein (ULBP) 1/2/3 and MHC class I polypeptide-related sequence (MIC) A/B, which reduces their recognition by endogenous NK cells^12^. Several studies have found that tumour cells alter metabolism to suppress NK cells or T cells by upregulation of indoleamine 2,3-dioxygenase enzymes^13,14^. Thus, tumour cells use various tools to escape antitumoral immunity in their hosts, and this immunological characteristic of tumour cells results in poor prognosis and malignancy.

Anisomycin is an antibiotic produced by *Streptomyces griseolus*^15,16^. Interestingly, anisomycin has been shown to inhibit eukaryotic protein synthesis and DNA synthesis, and has been reported to exert direct killing effects on several types of tumour cells^17,18^. However, the mechanisms underlying the immune-associated antitumoral effects of anisomycin have not been investigated.

In this study, we found the NK cell-mediated immunotherapeutic capacity on HCC was enhanced by treatemtn of anisomycin from *in vitro* cell based experiment and *in vivo* mouse model for the first time. To understand the mechanisms through which anisomycin mediates NK cell mediated antitumour effects, we performed a genome-scale analysis of gene expression profiles. We found that anisomycin treatment of HCC differentially regulated a broad range of immune regulation-associated genes. Among these immune regulation-associated genes, lymphocyte function-associated antigen-3 (LFA-3, also called *CD58*) was shown for its critical role to boost NK cell-mediated immunoregulatory effect on anisomycin treated HCC. Furthermore several immune associated proteins such as major histocompatibility complex molecules class I (MHC-I) and other adhesion molecule, ICAM4 on HCC cells were also regulated by treatment of anisomycin, which were considered as important for immune synapse formation between NK cells and HCC cells. The present findings provide important insights into the antitumoral mechanisms of anisomycin in HCC. Our investigation of the immune associated anti-tumor mechanism suggests is another important mechanism of anisomycin against HCCs through enhancement of immune synapse formation between NK cells and HCC cells in addition to direct killing effects of anisomycin on HCC cells. Thus, our findings provided important insights into the antitumor mechanisms of anisomycin in HCC.

## Results

### Anisomycin acts as a potent direct cytotoxic agent against HCC cells

**Figure 1:**
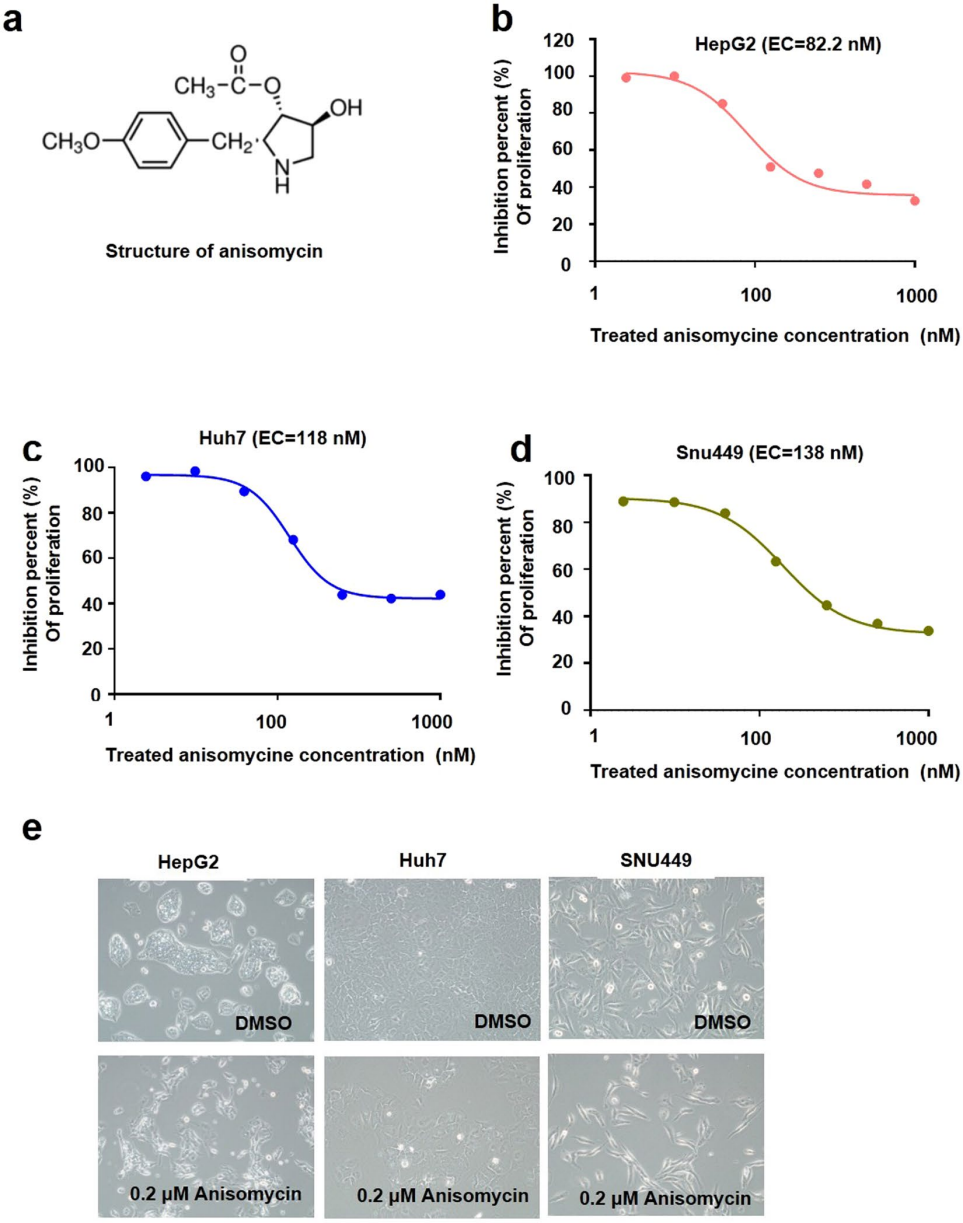
Inhibitory effects of anisomycin on HCC cells. (**a**) Anisomycin chemical structure; percent cell proliferation inhibition caused by anisomycin treatment in (**b**) HepG2, (**c**) Huh7, and (**d**) SNU449 cells was determined, and data were pooled from three independent experiments. The indicated IC_50_ values were estimated from the concentration-response curve. (**e**) HepG2, Huh7, and SNU449 cells were treated with DMSO (control) or 0.2 µM anisomycin for 48 h, and photographs were taken under a light microscope. Representative images from more than three independent experiments are shown.

Although the antitumoral cytotoxic effects of anisomycin (see chemical structure in Fig. 1a) have been previously reported^17,18^, the direct cytotoxic capacity of anisomycin in HCC cells has not been described to date. Thus, we investigated the cytotoxicity of anisomycin in multiple HCC cell lines. To assess this, we measured the direct effects of anisomycin on the viability and proliferation of HepG2, Huh7, and SNU449 cells using EZ-Cytox Enhanced cell viability assays. The half-maximal inhibitory concentration (IC_50_) values for anisomycin-mediated cell cytotoxicity were calculated from the respective concentration-response curves. Importantly, HepG2, Huh7, and SNU449 cells were all sensitive to anisomycin (IC_50_ values for HepG2, Huh7, and SNU449 cells: 82.2, 118, and 138 nM, respectively; Fig. 1b–d). Our results suggested that anisomycin was highly effective at inhibiting HCC cells. To confirm the cytotoxic effects of anisomycin, we examined HepG2, Huh7, and SNU449 cells after culture with 0.2 μM anisomycin for 2 days. We found that anisomycin reduced the cell densities of HepG2, Huh7, and SNU449 cell lines, as observed by light microscopy (Fig. 1e).

### Anisomycin triggered selective expression of genes associated with apoptosis and the immune response in HCC cells

**Figure 2:**
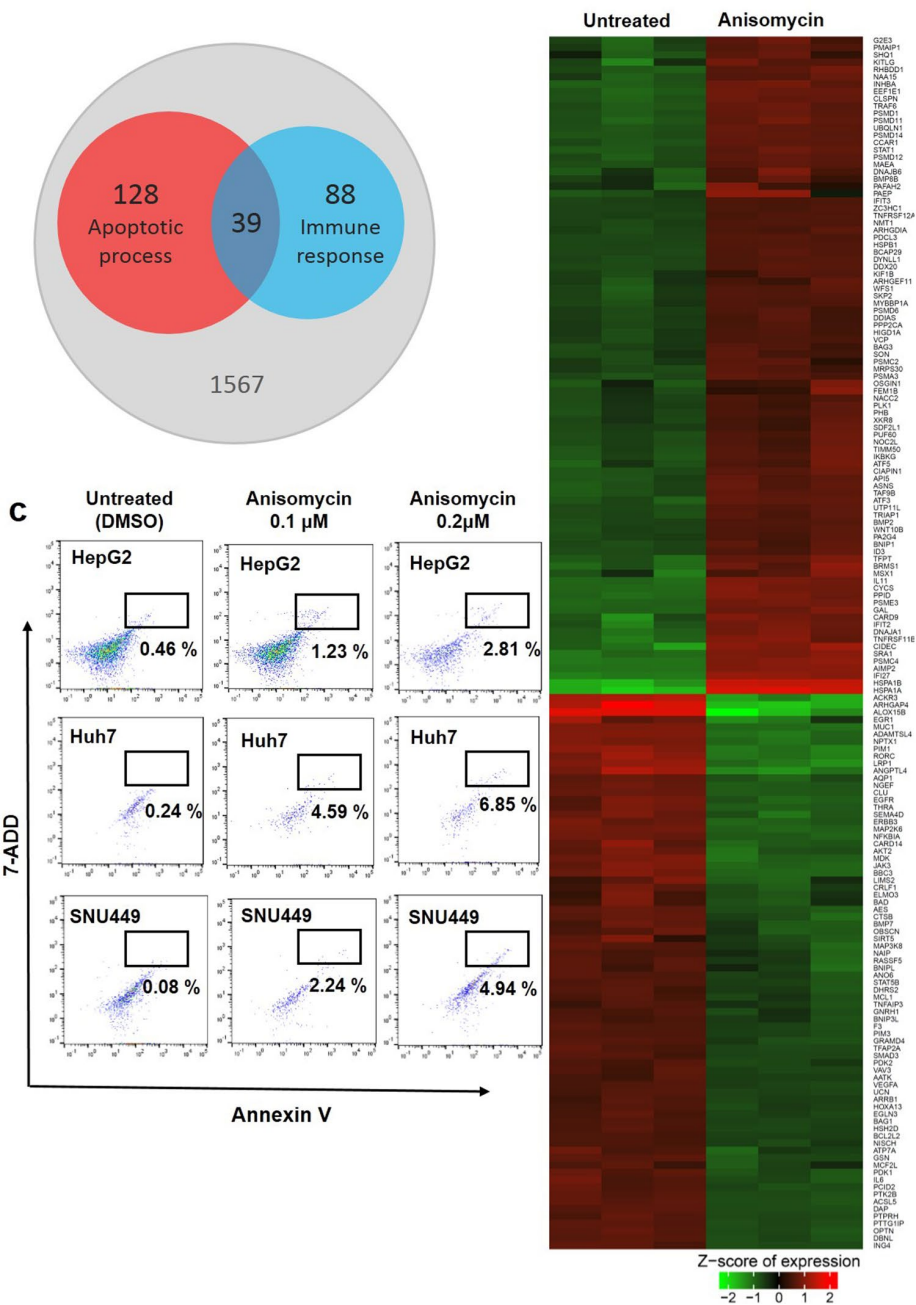
Anisomycin triggered apoptosis and regulated genes associated with apoptosis in HCC cells. (**a**) Venn diagrams displaying the number of differentially expressed genes associated with apoptotic process and immune response regulated in HepG2 cells after anisomycin treatment. Among 1821 genes applied for DAVID functional annotation analysis, 166 and 127 genes were identified as the associated genes for apoptotic process and immune response, respectively. And 39 genes were included in both sides. (**b**) Heat map representation of the expression levels of apoptosis-associated genes that were changed by more than 2-fold after anisomycin treatment of HepG2 cells in three independent experiments. The gene list for this heat map is shown in Supplementary Table 1. (**c**) HepG2, Huh7, and SNU449 cells were pre-treated with DMSO (control), 0.1 and 0.2 μM anisomycin for 48 h, and apoptosis was analysed by flow cytometry. Representative dot plots showing the percentage (%) of Annexin V and 7-ADD double-positive cells (apoptotic cells) from three independent experiments.

To better understand the antitumoral mechanisms of anisomycin, we performed a genome-scale analysis of gene expression profiles. To this end, we carried out microarray analysis to investigate transcriptional changes in HepG2 HCC cells, following 2 days of treatment with 0.2 µM anisomycin. We then compared the results with those in cells treated with vehicle (dimethyl sulphoxide [DMSO]). The analysis identified 1,173 probes that were upregulated by ≥2-fold, and 1,195 probes that were downregulated by at ≥2-fold in anisomycin-treated versus vehicle-treated HepG2 cells. To elucidate the genome-wide biological significance of these data, we selected and analysed the differentially expressed genes by functional annotation analysis using the DAVID database^19,20^. Probes which have their own gene ID were selected for the analysis. In the cases of duplicated genes with different probe ID, average of expression values of the duplicated genes was used for further study. Total 1828 genes were left after removing probes without gene ID and the duplicated genes. After excluding 7 genes as not identified in the DAVID database, 1821 genes were finally applied for functional annotation analysis. As a result, we found that anisomycin treatment on HCC differentially regulated broad range of apoptotic process-associated and immune response-associated genes (Fig. 2a). Total 166 apoptotic process-associated genes and 127 immune response-associated genes were differentially expressed by treating anisomycin. Thirty-nine genes were included in both annotations.

### Anisomycin induced apoptosis in HCC cells

The heat map of apoptosis-associated genes (Fig. 2b) showed that the expression of 90 genes was upregulated, while that of 76 genes was downregulated (listed in Supplementary Table 1). Thus, we attempted to confirm whether anisomycin induced apoptotic cell death in HCC cells. First, we stained anisomycin-treated HepG2 cells with Annexin V and 7-ADD, and then performed flow cytometry. We found that the proportion of apoptotic HepG2 cells (Annexin V-positive and 7-ADD-positive) increased in anisomycin concentration-dependent manner (Fig. 2c). Similar effects on apoptosis were also observed in Huh7 and SNU449 cells after treatment with anisomycin at the concentrations indicated in Fig. 2c.

### Anisomycin enhanced NK cell cytotoxicity in HCC cells *in vitro*

**Figure 3:**
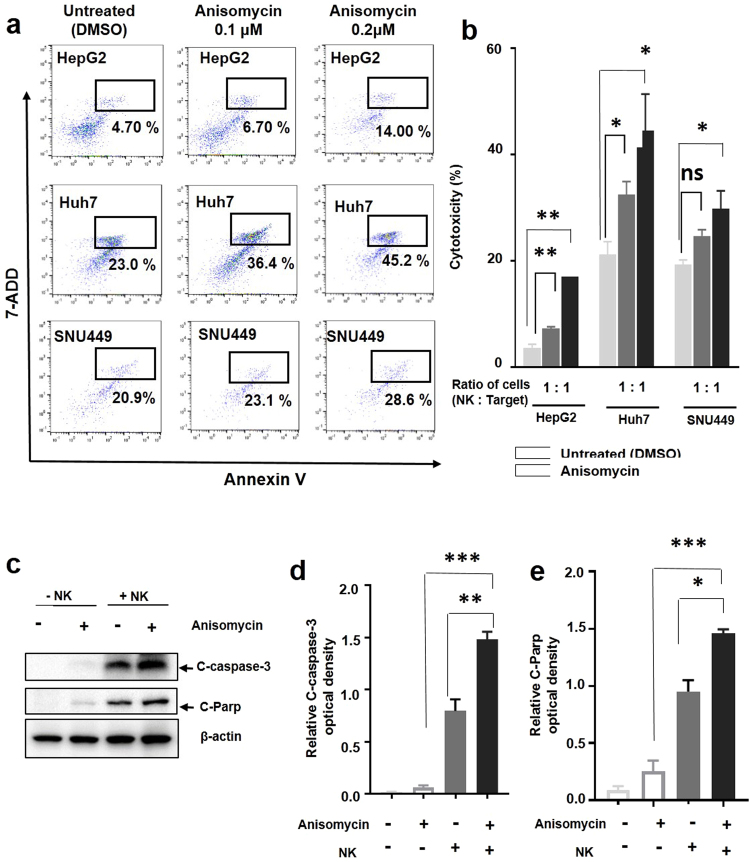
Anisomycin enhanced apoptosis-dependent NK cell cytotoxicity in HCC cells. (**a**) HepG2, Huh7, and SNU449 cells were pre-treated with DMSO (control), 0.1, and 0.2 μM anisomycin for 48 h and then cocultured with NK cells for 4 h. Apoptosis was analysed by flow cytometry. HepG2, Huh7, and SNU449 cells (CD56 negative) were gated with CD56 staining. Representative dot plots show the percentage (%) of Annexin V and 7ADD double-positive cells (apoptotic cells) from three independent experiments. (**b**) Cell cytotoxicity in cocultures of HepG2, Huh7, or SNU449 cells with NK cells. HepG2, Huh7, and SNU449 cultures were pre-treated with anisomycin or DMSO; pooled results are shown from three independent flow cytometry experiments; *^,^**significant differences from control (untreated) cells based on two-tailed unpaired Student’s t-tests at *p* < 0.05 and *p* < 0.01, respectively. The error bars indicate SEMs. The ratios of NK to HepG2, SNU449, and Huh7 cells are indicated. (**c**) HepG2 cells were pre-treated with DMSO (control) and 0.2 μM anisomycin for 48 h, cocultured with (+NK) or without NK cells (−NK) for 2 h, and then sorted into CD56-negative HepG2 cells. Protein expression in the cell lysates was analysed by immunoblotting with antibodies against cleaved caspase3 and cleaved PARP. β-Actin expression was analysed as a loading control. Full-length blots are presented in Supplementary Fig. 1. The results shown are representative of three independent experiments. (**d**) The densities of cleaved caspase3, and cleaved PARP were measured and normalized to β-actin levels. The relative level of cleaved caspase3 and cleaved PARP are shown. Pooled results are shown from three independent experiments; *^,^**^,^*** significant differences from the control (untreated) based on two-tailed unpaired Student’s t-tests at *p* < 0.05, *p* < 0.01, and *p* < 0.001, respectively. Error bars denote SEMs.

As described above, anisomycin treatment of HCC differentially regulated a broad range of immune response-associated genes, which accounted for about 7% of all differentially regulated genes affected by anisomycin (Fig. 2a). Thus, we next attempted to elucidate the mechanisms by which anisomycin enhanced the killing capacity of NK cells against HCC cells using *in vitro* experiments. In this study, we hypothesized that the NK cell-mediated enhanced antitumoral effects of anisomycin on HCC cells were caused by increased susceptibility of HCC cells to NK cell killing due to anisomycin-mediated changes in the expression of various HCC-related genes. To test this hypothesis, we pre-treated HepG2 cells with anisomycin for 2 days and then analysed the cytotoxicity of human primary NK cells isolated from peripheral blood on HepG2, Huh7, and SNU449 cells after removal of anisomycin. Interestingly, anisomycin-treated HepG2, Huh7, and SNU449 cells showed significant increases in susceptibility to NK cell killing compared with untreated HepG2, Huh7, and SNU449 cells (Fig. 3a,b). NK cells converted only 4.70% of HepG2 cells into the apoptotic state without anisomycin treatment; however, 14.00% of HepG2 cells that had been treated with 0.2 μM anisomycin were converted by NK cells (Fig. 3a). Similar effects were observed for Huh7 and SNU449 cells following anisomycin treatment (Fig. 3a,b).

To further confirm these findings, we analysed the protein levels of total caspase-3, cleaved caspase-3, poly (ADP-ribose) polymerase (PARP), and cleaved PARP, which are terminal effector molecules for induction of apoptosis. We compared these protein levels among untreated (DMSO), anisomycin-treated, NK cell-treated, and anisomycin plus NK cell-treated HepG2 cells. Importantly, we found that levels of the cleaved form of caspase-3 significantly increased following anisomycin treatment, with or without NK cell treatment (Fig. 3c). The level of cleaved caspase-3 protein was dramatically increased after exposure of HepG2 cells to NK cells (Fig. 3c,d). Notably, anisomycin treatment of HepG2 cells synergistically increased the cleaved caspase-3 protein after exposure to NK cells (Fig. 3c,d). As a result, cleaved PARP was also substantially increased by treatment with anisomycin and NK cells; these findings were similar to the observed patterns of cleaved caspase-3 expression (Fig. 3c,d). These data strongly suggest that a common mechanism underlies the enhancement of the susceptibility of HCC cells to NK cell killing, which may be triggered by anisomycin.

### Anisomycin regulated the expression of NK cell cytotoxicity-related molecules, such as CD58 and MHC-I, ICAM4 in HCC cells

**Figure 4:**
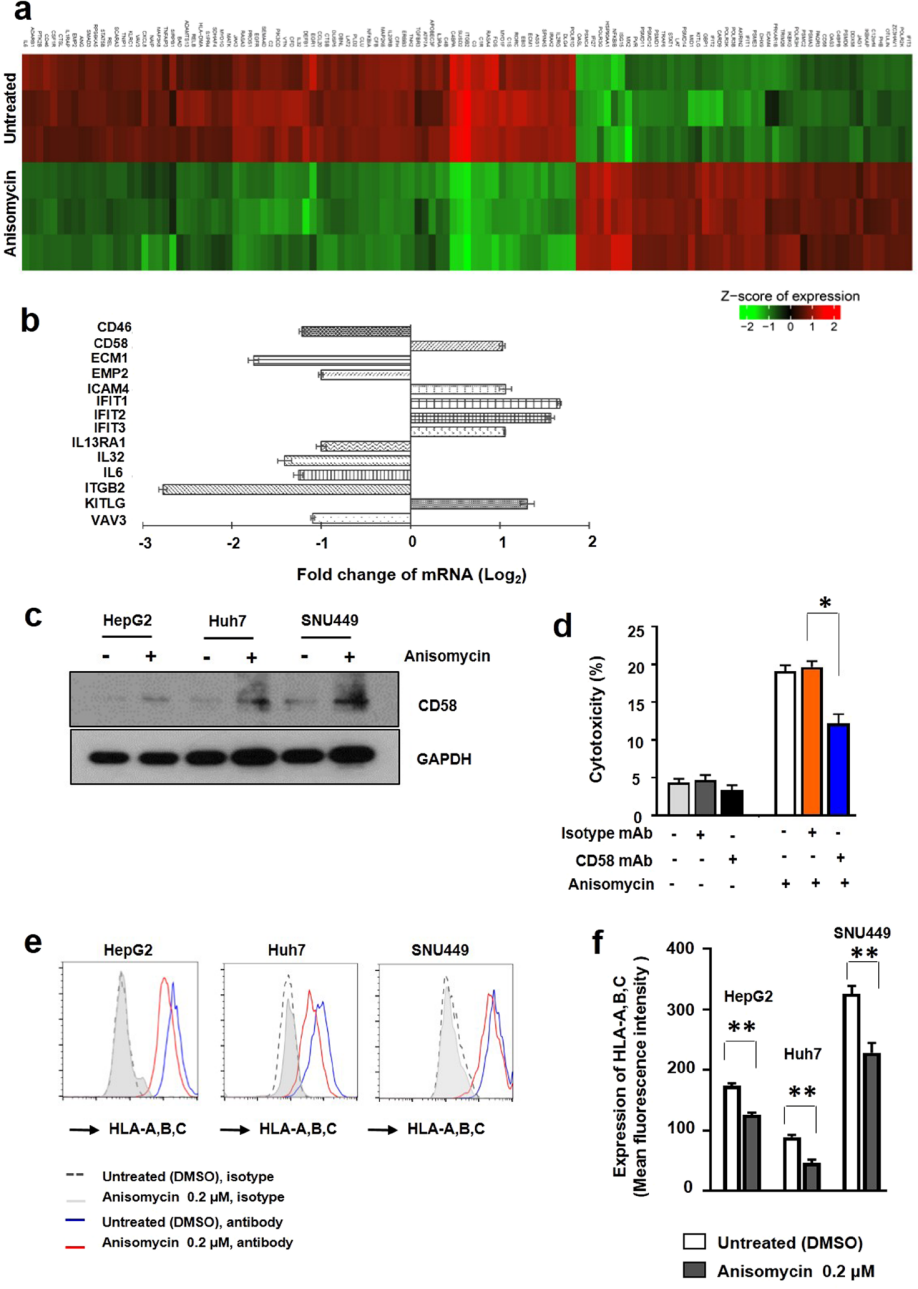
Anisomycin triggered selective regulation of genes associated with immune response-associated genes in HCC cells and the CD58 ligand-dependent effects of anisomycin on HCC cells toward NK cell cytotoxicity. (**a**) Heat map representation of the expression levels of immune response-associated genes that were changed by more than 2-fold after anisomycin treatment of HepG2 cells in three independent experiments. The gene list for this heat map is shown in Supplementary Table 2. (**b**) Selected differentially regulated genes in immune response-associated genes from microarray data. (**c**) HepG2, Huh7, and SNU449 cells were treated with DMSO (control) or 0.2 μM anisomycin and analysed to determine the expression levels of CD58 using western blotting. Protein expression in the cell lysates was analysed by immunoblotting with antibodies against CD58, and GAPDH expression was analysed as a loading control. Full-length blots are presented in Supplementary Fig. 2. The results shown are representative of three independent experiments. (**d**) HepG2 cells were pre-treated with DMSO (control) or 0.2 anisomycin for 48 h and then cocultured with NK cells for 4 h. NK cell cytotoxicity was measured using an LDH cytotoxicity assay kit with or without anti-CD58 blocking antibodies. The ratio of NK cells to HepG2 cells was 1: 1; * significant differences from the control (untreated) based on two-tailed unpaired Student’s t-tests at *p* < 0.05. Error bars denote SEMs. (**e**) HepG2, Huh7, and SNU449 cells were pre-treated with DMSO (control) or 0.2 μM anisomycin for 48 h and analysed by flow cytometry. Representative histograms are shown from three different independent experiments. (**f**) Pooled results are shown from three independent flow cytometry experiments; *^,^** significant differences from the control (untreated) by two-tailed unpaired Student’s t-tests at *p* < 0.05. Error bars denote SEMs.

We next investigated the anisomycin-mediated mechanism in HCC cells by analyzing genome-wide transcriptional changes in anisomycin-treated HepG2 cells (Fig. 4a). Based on our microarray data analysis, we first found that the expression of 48 genes was upregulated and that of 79 genes was downregulated in association with immune responses (Fig. 4a, listed in Supplementary Table 2). Among these genes, we aimed to identify genes whose alterations might affect NK cell cytotoxicity in HCC cells and found that CD58, ICAM4, Interferon-induced protein (IFIT)1, IFIT2, IFIT3 and KIT ligand (KITLG) were significantly up-regulated and CD46, ECM1, EMP2, and ITGB2 were down-regulated in anisomycin-treated HepG2 cells via a comparative analysis of previously reported data (Fig. 4b). Specifically, we focused on CD58 among those those genes because of association of CD58 with NK cell cytotoxicity based on previous reports^21,22^, demonstrated that CD58 was increased in anisomycin-treated HepG2, SNU449, and Huh7 cells and performed its role to boost susceptibility to NK cell cytotoxicity in the three different HCC cells (Fig. 4c,d). For this purpose, we measured the protein expression levels of CD58 in HepG2, SNU449, and Huh7 cells. For this experiment, HepG2, Huh7, and SNU449 cells were cultured in the absence or presence of 0.2 μM anisomycin. Total protein was extracted from the cells, and the level of CD58 was analysed by western blotting (Fig. 4c). Notably, anisomycin increased the expression of CD58 in all three HCC cell lines (Fig. 4c). To functionally assess the role of CD58 in NK-mediated killing of HCC cells, we measured NK-induced cell cytotoxicity in cocultures with HepG2 cells with or without anisomycin, along with blockade of CD58 using an anti-CD58 antibody after anisomycin treatment. Cell cytotoxicity was assessed using a lactate dehydrogenase (LDH) cytotoxicity assay kit. Notably, blockade of CD58 significantly reduced anisomycin-mediated enhanced NK cytotoxicity (Fig. 4d). Additionally, genes encoding several critical cytokines, such as interleukin (IL)-32 and IL-6, as well as cytokine response-associated signalling proteins such as interferon-induced protein with tetratricopeptide repeats (IFIT) 1, IFIT2, IFIT3, and IL-13RA1, were also significantly regulated by anisomycin in HCC cells (Fig. 4b). We additionally found that HLA-DMA, a type of MHC-II, was significantly downregulated by anisomycin in HCC cells (Fig. 4a and Supplementary Table 2). Similarly, MHC-I molecules, such as HLA-A, -B, and -C, were significantly downregulated by anisomycin at the protein level in HepG2, SNU449, and Huh7 cells, as demonstrated using flow cytometry (Fig. 4e,f). MHC-I molecules are known for their inhibitory activity against NK cell killing on tumour cells^23^. Thus, anisomycin-mediated downregulation of MHC-I may increase the susceptibility of HCC cells to NK cell killing. Taken together, anisomycin treated HCC cells regulated expression of adhesion molecules, CD58, MHC-I, and ICAM-4 to strengthen interaction of NK cells to HCC celles, which caused NK-mediated killing of HCC cells as we confirmed in Figs 3a and 4d.

### Anisomycin enhanced NK cell cytotoxicity in HCC cells in a HepG2 carcinoma-bearing SCID mouse model

**Figure 5:**
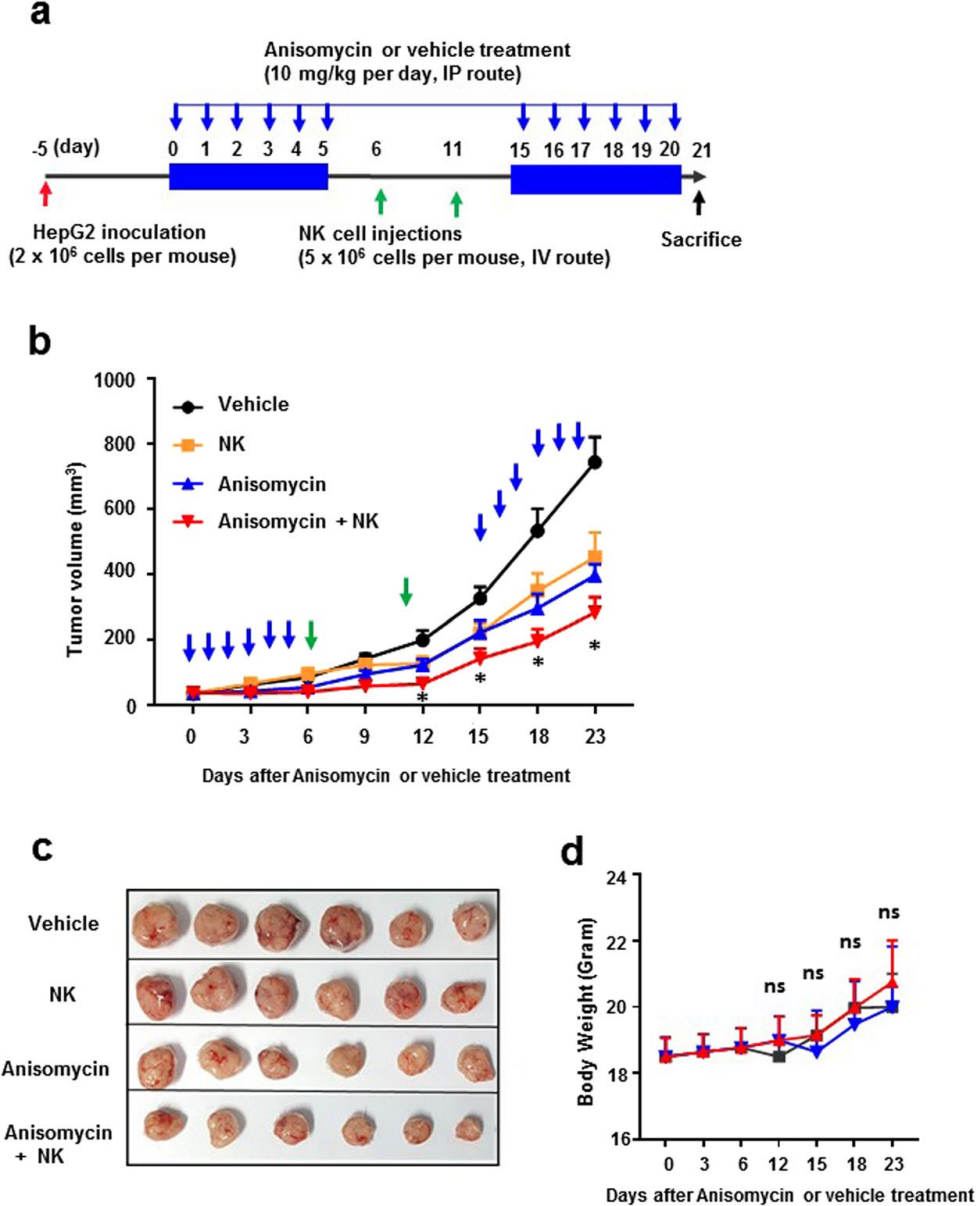
NK cell-dependent effects of anisomycin in an HCC xenograft mouse model. (**a**) Schematic plot of the study design and route of injection for therapeutic efficacy. (**b**) Five days after inoculation of HepG2 cells, anisomycin (10 mg/kg) was administered from days 0 to 5 and from days 15 to 20 after initiation of treatment via the intraperitoneal (i.p) route. NK cells (5 × 10^6^ cells/mouse) were transferred into mice two times on days 6 and 11 during the treatment pause period, as described in the Materials and Methods (n = 6 mice). Tumour sizes were measured on the indicated days. (**c**) Tumours in each group of mice (n = 6) on day 23 of the experiment. (**d**) Body weights of each group of mice (n = 6) were measured every 3 days.

As described above, anisomycin induced differential expression of immunomodulatory genes, particularly those related to the functional regulation of NK cells, thus indicating the NK cell-mediated anti-HCC effects of anisomycin. As previously reported^24^, NK cells are the most potent antitumoral innate immune cells and are major target cells for immune escape by various cancers. We hypothesized that anisomycin may regulate the susceptibility of HCC cells to NK cell-mediated antitumoral immune responses. To investigate the NK cell-mediated changes in HCC cells triggered by anisomycin, we attempted to eliminate the potential effects of other potent antitumoral immune cells, specifically T cells. NOD-SCID mice lack T cells and NK cells and thus do not develop an adaptive immune system or NK-mediated immune responses. Therefore, we generated an HCC tumour xenograft model in NOD-SCID mice using HepG2 cells with or without transfusion of human primary NK cells isolated from peripheral blood, to investigate the effects of anisomycin on NK cell-mediated antitumoral immunity. Specifically, to elucidate whether the effects of anisomycin on tumour suppression in the HepG2 carcinoma mouse model were mediated by NK cells, we compared the growth of HepG2 carcinomas in mice with and without intravenous injection of human NK cells plus anisomycin treatment. For this purpose, we designed an *in vivo* study, as detailed in the scheme in Fig. 5a. Notably, we found that anisomycin significantly reduced HepG2 tumour size in mice, as shown in Fig. 5b. More importantly, tumour suppression by anisomycin was synergistically enhanced in the presence of human primary NK cells (Fig. 5b,c). These results strongly suggest that NK cells played a critical role in the antitumoral effects of anisomycin in HCC. During the experiments, anisomycin-treated mice did not show significant body weight loss or any abnormal behaviours (Fig. 5d).

## Discussion

Anisomycin, a natural antibiotic isolated from *S*. *griseolus*^15,16^, exerts inhibitory effects in eukaryotic ribosomes, blocking the activity of peptidyl transferase^24^. These activities of anisomycin in eukaryotes may affect various cellular functions such as regulation of protein expression, survival and proliferation^18,25,26^. Moreover, the anisomycin-mediated effects may be mediated by regulation of intracellular signalling pathways such as the mitogen-activated protein kinase (MAPK) pathway^26^, decreasing the caspase inhibitor, c-Fas-associated death domain-like interleukin-1 (IL-1)-converting enzyme-like inhibitory protein (FLIP) and activating death receptor pathway^27,28^, and activation of c-Jun and p-38 kinase^29,30^. Clearly, anisomycin triggers multiple anti-cancer associated signalling in various cancer cells including HCC cells. The signalling pathways triggered by anisomycin might be different among different cells due to differentiated intracellular signalling scaffolds, which vary among different cells. Although it is not fully understood yet, anisomycin clearly affect cell death signal.

Based on our genome-wide transcriptional analysis using the Kyoto Encyclopedia of Genes and Genomes tool, we also found that multiple signalling pathways, including the MAPK, tumour necrosis factor receptor, and nuclear factor kappaβ signalling pathways, may be affected by anisomycin in HCC cells (data not shown). These effects of anisomycin on intracellular signalling pathways may induce apoptosis and suppress growth of HCC cells. Indeed, our results showed that anisomycin exerted potent growth inhibitory effects or direct cytotoxicity in multiple HCC cell lines; further, these inhibitory effects of anisomycin may be caused by the induction of apoptosis in HCC cells. Thus, alterations in multiple intracellular signals triggered by anisomycin may explain cellular apoptosis and cytotoxicity in HCC cells.

More importantly, we first showed anisomycin-mediated sensitizing effect on HCC cells in this study. We found that the antitumoral effects of anisomycin on HCC cells were synergistically enhanced by regulation of NK cell killing capacity. This is the first report for NK-mediated anti-cancer effect of anisomycin until now. To understand this immunotherapeutic effect of anisomycin, we performed genome-wide transcriptional analysis and found that anisomycin treatment of HCC cells regulated a broad range of immune-associated genes, including multiple immune-associated genes. Among these genes, the expression levels of *CD58* and *ICAM4*, which are known for their roles in intercellular adhesion, were increased more than 2-fold. ICAM4 binds to integrin proteins, such as CD11b/CD18 and CD11b/CD18^31^, indicating a role in the interactions between immune cells and HCC cells. CD58 is involved in intercellular adhesion between NK cells and tumour cells by binding to CD2 on NK cells^22^, suggesting that the anisomycin-mediated increase in CD58 and ICAM4 enhanced the strengthen immune synapse formation between NK cells and anisomycin treated HCC cells causing increased susceptibility of HCC cells to NK cell killing. Thus, we confirmed our hypothesis using an anti-CD58-neutralizing antibody in this study (Fig. 4d). Our findings supported that anisomycin-mediated regulation of CD58 in HCC cells might affect the T-cell response, as CD2 is also expressed on T cells^21^. Thus, we hypothesized that anisomycin triggered T cell-mediated antitumoral effects. Further studies of the adjuvant effects of anisomycin on T cell-mediated antitumoral activities are needed.

Additionally, we found that several critical proteins, including MHC-I molecules (HLA-A, -B, and -C), were significantly downregulated by anisomycin treatment in all three HCC cell lines. Changes in the expression levels of these proteins were not found in our genome-wide transcriptional analysis; however, we found that the expression of a similar antigen presentation-associated gene, *HLA-DMA* (a type of MHC-II), was also significantly reduced. Based on a previous study^23^, MHC-I molecules, as ligands for Ly49 receptors on NK cells, inhibit the killing of tumour cells expressing self-MHC-I. Thus, reduction of MHC-I molecules clearly provides advantages for NK cells to recognize and kill tumour cells. Our findings that anisomycin decreased MHC-I expression and enhanced the susceptibility of HCC cells to NK cell killing provided insights into the mechanisms by which anisomycin boosts NK activity in tumour cells *in vitro* and tumour tissues *in vivo*. Furthermore, we identified other immune-associated genes in HCC cells that were regulated by anisomycin. For example, the *IFIT1*, *IFIT2*, and *IFIT3* genes were significantly upregulated by anisomycin, and may have roles in interferon-α-triggered cellular growth arrest and apoptosis^32^. Although IFIT1, IFIT2, and IFIT3 exert critical antitumoral effects, it is unclear how anisomycin induces upregulation of these proteins. Therefore, further studies are needed to determine the mechanisms underlying the induction of IFIT1, IFIT2, and IFIT3, and to elucidate how these proteins function in HCC.

Among immune-associated genes, we also found that the *CD46*, *Vav3*, *ITGB2*, *EMP2*, and *ECM1* genes in HepG2 cells were downregulated by anisomycin; these genes were previously reported to have functions in cellular metastasis in various cancer cell lines^33–36^. Another important group of immune-associated genes regulated by anisomycin included *KITLG*, *IL-6*, *IL-32*, and *IL-3RA*, which encode cytokines and cytokine receptors to mediate potential survival signals or growth signals in tumour cells. Importantly, most cytokine and cytokine receptor genes were downregulated by anisomycin treatment in HepG2 cells. The downregulation of cytokines and cytokine receptors may suppress the functions of cytokines or other growth factors, triggering survival signals synergistically with reduction of cytokine-related intracellular signalling pathway genes such as *STAT5B*, *IRAK2*, *RELB*, and *JAK3*.

Compared with previous studies, which focused on the cytotoxic and inhibitory effects of anisomycin on HCC cells^27^, our study demonstrated, based on genome-wide transcriptional analysis, that anisomycin exerts immunomodulatory effects by regulating the expression of a broad range of genes in HCC cells; these findings were confirmed both *in vitro* and *in vivo*. Our study suggested that anisomycin elicited antitumoral effects in an immune-mediated manner; these effects were specifically dependent on its indirect stimulatory effects on NK cells, in addition to direct regulation of tumour cell growth or apoptosis. Also, in our data (Fig. 3a,b), we found that the increase in apoptotic effect upon co-culture with NK cells on HepG2 cells was much more pronounced comparison with Huh7 and SNU449 cells. This result might suggest that there are differences among the anisomycin-triggered molecular sensitizing characteristics of different HCC cells for NK cell cytotoxicity. While we could not clearly understand what make differences in the apoptotic effect upon co-culture effect with NK cells on different HCC cells yet, it might be possible that a combined effects of multiple molecules such as CD58, ICAM-4, MHC-I (HLA-A,B,C) which were differentially regulated by anisomycin in different cells could make HepG2 cells to be more sensitive for NK cell cytotoxicity in comparison to SNU449 and Huh7. Therefore, our study suggested that the immune-mediated antitumoral effects of anisomycin represent another important mechanism through which anisomycin eliminated HCC cells despite our incomplete understanding. Further, the findings show that the novel immunotherapeutic effects of anisomycin may represent a new strategy for the development of effective therapeutic agents for HCC.

## Methods

### Mice

NOD.CB17-Prkdc^scid^/NCrCrl mice were purchased from Charles River Laboratories, Inc. (Wilmington, MA, USA). We used 6-8-week-old female mice in all experiments; these mice were housed in a pathogen-free animal facility. All animal experiments were approved by the Institutional Animal Use and Care Committee of the Korea Research Institute of Bioscience and Biotechnology and performed in accordance with the Guide for the Care and Use of Laboratory Animals published by the US National Institutes of Health.

### Xenograft study

Six-week-old female NOD.CB17-Prkdc^scid^/NCrCrl mice were purchased from Charles River Laboratories, Inc. Xenograft models were established by subcutaneous injection of 1 × 10^6^ HepG2 cells into the right flanks of the mice. Five days after inoculation, the mice were randomly separated into groups of four. Anisomycin (10 mg/kg) was administered from day 0 to 5 and from day 15 to 20 after initiation of anisomycin treatment via the intraperitoneal (i.p) route. NK cells (5 × 10^6^ cells/mouse) were transferred into mice two times on days 6 and 11 during the treatment pause period. Tumours were measured every 3 days using callipers, and their volumes were calculated according to the following formula: volume (mm^3^) = (d^2^ × D)/2, where d and D represent the shortest and longest tumour diameters, respectively.

### Human peripheral blood and NK cell sorting

Peripheral blood from healthy donors was obtained from the Red Cross Blood Center. We followed the guidelines of the Red Cross, and all methods and protocols involving the use of human peripheral blood were approved by the Institutional Review Board of the Red Cross. Based on the Red Cross guidelines, informed consent for study participation was obtained, and donor information was not provided. NK cells were isolated from whole blood to more than 95% purity by negative selection using a RosetteSep Human NK Cell Enrichment Cocktail (StemCell Technologies, Vancouver, Canada) according to the manufacturer’s protocol. The isolated NK cells were washed in HBSS plus 4% foetal bovine serum (FBS; Life Technologies, Carlsbad, CA, USA) and resuspended in MEM-α (Life Technologies) containing 20% FBS (Life Technologies) and penicillin-streptomycin (Life Technologies) at 37 °C in 5% CO_2_.

### NK cell cytotoxic assay

Evaluation of NK cell activity was performed using an LDH Cytotoxicity Assay Kit (Thermo Fisher Scientific, Waltham, MA, USA). Cell suspensions (100 μL) from anisomycin-treated or untreated tumour cell lines (HepG2, SNU449, or Huh7; 1.0 × 10^6^ cells/mL) were added to 100 μL NK cells (8.0 × 10^6^ cells/mL, 4.0 × 10^6^ cells/mL, or 2.0 × 10^6^ cells/m) in RPMI 1640 medium (Life Technologies) to provide effector-to-target cell ratios (E:T) of 0.5: 1 and 1: 1, and the cells were incubated in 96-microwell plates (Life Technologies) for 4 h at 37 °C in a humid atmosphere containing 5% CO_2_. Subsequently, the plates were centrifuged for 5 min at 200 × *g*; then, 100 μL of supernatant from each well was transferred to 96-well flat-bottomed plates, and 100 μL of lactic acid dehydrogenase substrate mixture was added. Colour development (5 min in the dark) was measured at 492–630 nm using a microtiter plate reader (TECAN, Melbourne, Australia). The mean cytotoxicity percentage was calculated as follows: (LDH experimental − LDH spontaneous − LDH from NK cells)/(LDH maximal − LDH spontaneous) × 100 (%). LDH experimental release represented the LDH release from NK cell and tumour cell cocultures. Spontaneous LDH release activities from tumour cells and NK cells were obtained from individual cultures. The maximal release of LDH activity was obtained following lysis of tumour cells, using ultrasound (Soniprep 1500; Behring, Germany). For neutralization experiments, anti-human CD58/LFA-3 monoclonal antibodies (R&D Systems, Minneapolis, MN, USA) were used to pre-treat target cells (HepG2 cells) for 20 min before coculture with NK cells at a ratio of 1: 1, and evaluation of NK cell activity was performed using an LDH Assay Kit, as described above.

### Cell culture and reagents

HepG2 cells were purchased from the American Type Culture Collection (ATCC, Manassas, VA, USA), and Huh7 cells and SNU449 cells were purchased from the Korean Cell Line Bank (Seoul, Korea). Cells were cultured according to the suppliers’ instructions. HepG2 cells were cultured in Eagle’s minimum essential medium (ATCC) containing 10% FBS (Life Technologies), 2 mM l-glutamine, and penicillin-streptomycin (Life Technologies) at 37 °C in 5% CO_2_. Huh7 and SNU449 cells were cultured in RPMI1640 (Life Technologies) containing 10% FBS (Life Technologies), 2 mM l-glutamine, and penicillin-streptomycin (Life Technologies) at 37 °C in 5% CO_2_. Anisomycin was purchased from Sigma-Aldrich (St. Louis, MO, USA) and dissolved at a concentration of 20 mg/mL in 100% DMSO as a stock solution. The stock solution was stored at −20 °C and diluted in medium before each experiment. The final DMSO concentration did not exceed 0.1% throughout this study (all control groups were administered 0.1% DMSO). Antibodies against caspase3, PARP, and β-actin were purchased from Cell Signaling Technology (Danvers, MA, USA).

### Microarray and data analysis

HepG2 cells (5 × 10^6^) in 10 mL of complete RPMI culture medium were incubated for 48 h at 37 °C with DMSO alone (control) or with 0.2 μM anisomycin. Total RNA was isolated from HepG2 cells using TRIzol (Invitrogen, Carlsbad, CA, USA). Analyses were conducted using an Affymetrix whole-genome expression microarray performed according to the manufacturer’s instructions. Data were exported and normalized with the robust multi-average method implemented in Affymetrix Expression Console software. Statistical significance was determined using the local pooled error test and gene expression fold change, in which the null hypothesis was that no difference existed among groups. The false discovery rate was controlled by adjusting the *P* values using the Benjamini-Hochberg algorithm. For differentially expressed genes, hierarchical cluster analysis was performed using complete linkage and Euclidean distance as a measure of similarity. Functional annotation analyses for the significant probe list were performed using DAVID (http://david.abcc.ncifcrf.gov/home). Before analysis by DAVID, genes that were duplicated or were not identified in the database were excluded. The analyses and visualization of differentially expressed genes using a heatmap were conducted using R software (www.r-project.org).

### Cell viability and proliferation assays

Cell proliferation was assessed using an EZ-Cytox Enhanced cell viability assay kit from ITSBIO (Seoul, Republic of Korea). Briefly, the appropriate number of cells was plated in each well of a 96-well plate and exposed to different concentrations of anisomycin for 48 h. Subsequently, the tetrazolium salt reagent was added, and the cells were incubated for 2 h. At the end of the incubation, the absorbance in each well was measured at 450 nm using a microplate reader. The relative cell viability (%) was calculated using the equation OD^T^/OD^C^ × 100% (where OD^T^ represents the absorbance of the treatment group, and OD^c^ represents the absorbance of the control group). The median inhibitory concentration (IC_50_) was defined as the drug concentration required to inhibit 50% of the cells relative to the controls. IC_50_ values were estimated from the concentration-response curve.

### Antibodies and flow cytometry

To analyse apoptotic cells, we stained cells with anti-human CD56-allophycocyanin monoclonal antibodies, 7ADD, and Annexin V (BioLegend, San Diego, CA, USA) following the protocol provided by the manufacturer. Briefly, we stained cells with anti-human CD56-allophycocyanin monoclonal antibodies on ice for 10 min, washed cells with an appropriate volume of chilled Annexin V binding buffer (BioLegend), and resuspended cells in chilled Annexin V binding buffer (BioLegend) in the presence of Annexin V and 7ADD. The cells were incubated on ice for 30 min, washed with chilled Annexin V binding buffer, and analysed. Staining data were collected using a FACSCanto II Cytometer (BD Biosciences).

### Cell sorting

HepG2 cells were cultured in the presence or absence of 0.2 µM anisomycin for 2 days and harvested for cell counts. Then, 1 × 10^7^ HepG2 cells were cocultured with 1 × 10^7^ NK cells for 2 h and harvested by centrifugation at 1,000 rpm for 5 min. Cells were stained with anti-human CD56 monoclonal antibodies (BioLegend), washed, and resuspended in PBS containing HBSS supplemented with 4% FBS (Gibco). CD56-negative HepG2 cells were isolated to nearly 100% purity using an Aria cytometer (BD Biosciences).

### Total RNA isolation and real-time reverse transcription polymerase chain reaction (qPCR)

Cells were prepared as described above. Total cellular RNA was isolated using TRIzol reagent (Invitrogen). Real-time RT-PCR was performed using RNA (20 ng) as the template, qScript cDNA SuperMix for the reverse transcription step, and PerfeCTa qPCR FastMix, UNG, and ROX for PCR (Quanta Biosciences, Gaithersburg, MD, USA). Primer and probe sets (FAM/VIC-labelled) were purchased from Applied Biosystems (Waltham, MA, USA). The results were normalized based on the values obtained for *GAPDH* as detected using TaqMan *GAPDH* control reagents (Applied Biosystems). Real-time qPCR was performed with samples in duplicate using the ABI 7700 Sequence Detection System (Applied Biosystems). For cells from each donor, relative expression levels based on 2^−ΔΔCT^ values are shown as percentages relative to values obtained for the subset with the highest expression.

### Western blot analysis

Cells were suspended in modified RIPA lysis buffer (150 mM NaCl, 1 mM EDTA, 1% Triton X-100, 1% NP-40, 0.5% sodium deoxycholate, 0.1% sodium dodecyl sulphate [SDS], and 50 mM Tris-HCl [pH 7.4]) containing a protease inhibitor cocktail (Roche, Mannheim, Germany) and phosphatase inhibitors (1 mM sodium fluoride and 2 mM sodium orthovanadate) on ice for 30 min and centrifuged at 15,000 × *g* for 30 min to collect whole-cell lysates. Proteins (10–20 µg) were then separated by SDS-polyacrylamide gel electrophoresis on 8‒12% gels and transferred to polyvinylidene difluoride membranes (Millipore, Bedford, MA, USA). Western blotting was performed with primary antibodies against caspase3, PARP, and β-actin (Cell Signaling Technology) and peroxidase-conjugated anti-mouse or anti-rabbit secondary antibodies. Proteins were visualized with Enhanced Chemiluminescence Plus reagents (Amersham Biosciences, Piscataway, NJ, USA).

### Statistical analysis

Analysis of variance was used for multiple comparisons. Unpaired *t* tests were performed to analyse differences between two groups. Statistical analyses were performed using the Prism software package (GraphPad Software, San Diego, CA, USA). Differences with *p* values of 0.05 or less were considered significant.

## Electronic supplementary material

Supplementary Information
