# Supplementary material for: Novel natural killer cell-mediated cancer immunotherapeutic activity of anisomycin against hepatocellular carcinoma cells
Source: Sci Rep. 2018 Jul 13;8:10668. doi: 10.1038/s41598-018-29048-8 (PMC6045618; doi:10.1038/s41598-018-29048-8)
Supplement: Supplementary file 1 — Supplementary Information [file 41598_2018_29048_MOESM1_ESM.pdf]

Supplementary Information

Novel natural killer cell-mediated cancer immunotherapeutic activity of anisomycin against hepatocellular carcinoma cells

Miok Kim, Seon-Jin Lee, Sangsu Shin, Kang-Seo Park, Sang Yoon Park and Chang Hoon Lee

Supplementary figure 1: Immunoblot analysis of cleaved caspase 3 and cleaved Parp in HepG2 cells treated with/without anisomycin and NK cells.

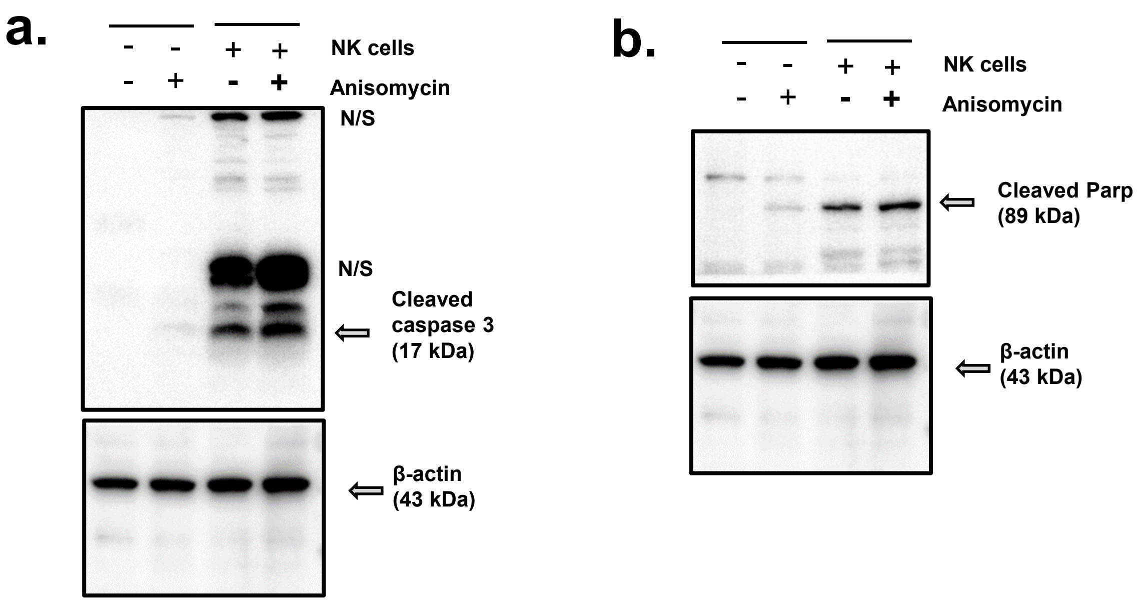

HepG2 cells were pre-treated with DMSO (control) and 0.2 μM anisomycin for 48 h, cocultured with (+NK) or without NK cells (-NK) for 2 h, and then sorted into CD56-negative HepG2 cells. Protein expression in the cell lysates was analysed by immunoblotting with antibodies against (a) cleaved caspase3 and (b) cleaved PARP. β-Actin expression was analysed as a loading control. N/S indicates non-specific band.

Supplementary figure 2: Immunoblot analysis of CD58 and GAPDH in HepG2, Huh7, SNU449 cells treated with/without anisomycin.

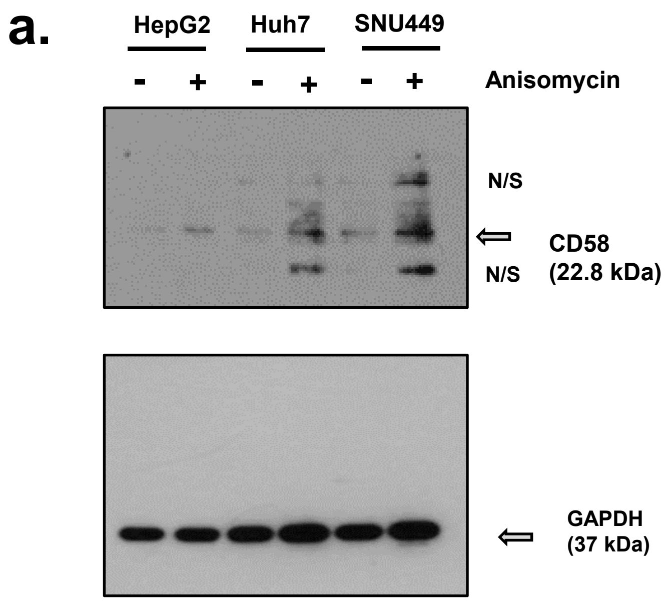

(a) HepG2, Huh7, and SNU449 cells were treated with DMSO (control) or 0.2 μM anisomycin and analysed to determine the expression levels of CD58 using western blotting. Protein expression in the cell lysates was analysed by immunoblotting with antibodies against CD58, and GAPDH expression was analysed as a loading control. N/S indicates non-specific band.

Supplementary Table 1. List of genes associated with apoptotic process and differentially regulated by anisomycin treatment in HepG2 cells.

Apoptotic process	
**Down-regulated Genes**	AATK, ACKR3, ACSL5, ADAMTSL4, AES, AKT2, ALOX15B, ANGPTL4, ANO6, AQP1, ARHGAP4, ARRB1, ATP7A, BAD, BAG1, BBC3, BCL2L2, BMP7, BNIP3L, BNIPL, CARD14, CLU, CRLF1, CTSB, DAP, DBNL, DHRS2, EGFR, EGLN3, EGR1, ELMO3, ERBB3, F3, GNRH1, GRAMD4, GSN, HOXA13, HSH2D, IL6, ING4, JAK3, LIMS2, LRP1, MAP2K6, MAP3K8, MCF2L, MCL1, MDK, MUC1, NAIP, NFKBIA, NGEF, NISCH, NPTX1, OBSCN, OPTN, PCID2, PDK1, PDK2, PIM1, PIM3, PTK2B, PTPRH, PTTG1IP, RASSF5, RORC, SEMA4D, SIRT5, SMAD3, STAT5B, TFAP2A, THRA, TNFAIP3, UCN, VAV3, VEGFA
**Up-regulated Genes**	AIMP2, API5, ARHGDIA, ARHGEF11, ASNS, ATF3, ATF5, BAG3, BCAP29, BMP2, BMP8B, BNIP1, BRMS1, CARD9, CCAR1, CIAPIN1, CIDEC, CLSPN, CYCS, DDIAS, DDX20, DNAJA1, DNAJB6, DYNLL1, EEF1E1, FEM1B, G2E3, GAL, HIGD1A, HSPA1A, HSPA1B, HSPB1, ID3, IFI27, IFIT2, IFIT3, IKBKG, IL11, INHBA, KIF1B, KITLG, MAEA, MRPS30, MSX1, MYBBP1A, NAA15, NACC2, NMT1, NOC2L, OSGIN1, PA2G4, PAEP, PAFAH2, PDCL3, PHB, PLK1, PMAIP1, PPID, PPP2CA, PSMA3, PSMC2, PSMC4, PSMD1, PSMD11, PSMD12, PSMD14, PSMD6, PSME3, PUF60, RHBDD1, SDF2L1, SHQ1, SKP2, SON, SRA1, STAT1, TAF9B, TFPT, TIMM50, TNFRSF11B, TNFRSF12A, TRAF6, TRIAP1, UBQLN1, UTP11L, VCP, WFS1, WNT10B, XKR8, ZC3HC1

Supplementary Table 2. List of genes associated with immune response and differentially regulated by anisomycin treatment in HepG2 cells.

**Immune response**	
**Down-regulated Genes**	ADAMTS13, ADARB1, ANG, APOBEC3F, ASS1, ATP7A, BAD, C1R, C1S, C2, C3, C4B, C4BPA, CCL20, CD46, CFB, CFD, CFH, CLU, CSF1R, CTSB, CTSL, CXCL8, DBNL, DEFB1, DLG4, DUSP5, EBI3, ECM1, EGFR, EGR1, EMP2, ERBB3, FOS, HLA-DMA, IL1RAP, IL20RB, IL2RG, IL32, IL3RA, IL6, IRAK2, ITGB2, JAK3, KLRC1, LAT2, MAP2K6, MAP3K8, MATK, MYO1F, MYO1G, NAIP, NFKBIA, PIK3CD, PLD2, POLR1D, PROS1, PTK2B, RASA3, RASA4, REL, RELB, RORC, RPS6KA5, S1PR4, SCARA3, SDHAF4, SEMA4D, SIRPB1, SMAD3, SPINK5, STAT5B, SUSD2, TGFBR3, TNFAIP3, TNIP1, TNK2, VAV3, VTN
**Up-regulated Genes**	AKIRIN2, C12ORF4, C4BPB, CARD9, CD58, DDX58, DHX9, GBP1, HSP90AA1, ICAM4, IFI27, IFIT1, IFIT2, IFIT3, IKBKAP, IKBKG, ISG15, JAG1, KITLG, LAT, MID1, MX2, NFKBIB, OAS3, OASL, OTULIN, PAQR3, PHB, POLR2K, POLR3B, POLR3G, POLR3H, POLR3K, PRKAR1B, PSMA3, PSMC2, PSMC4, PSMD1, PSMD11, PSMD12, PSMD14, PSMD6, PSME3, PVR, STAT1, TRAF6, TRIM26, ZC3HAV1
